# Plant Ureases and Related Peptides: Understanding Their Entomotoxic Properties

**DOI:** 10.3390/toxins4020055

**Published:** 2012-02-01

**Authors:** Fernanda Stanisçuaski, Célia R. Carlini

**Affiliations:** 1 Department of Molecular Biology and Biotechnology, Universidade Federal do Rio Grande do Sul, Av. Bento Gonçalves 9500, Porto Alegre, 91501-970, Brazil; 2 Department of Biophysics and Center of Biotechnology, Universidade Federal do Rio Grande do Sul, Av. Bento Gonçalves 9500, Porto Alegre, 91501-970, Brazil; Email: ccarlini@ufrgs.br

**Keywords:** plant defense, urease, Jaburetox-2Ec, Malpighian tubules, midgut, fluid secretion

## Abstract

Recently, ureases were included in the arsenal of plant defense proteins, alongside many other proteins with biotechnological potential such as insecticides. Isoforms of *Canavalia ensiformis* urease (canatoxin—CNTX and jack bean urease—JBURE-I) are toxic to insects of different orders. This toxicity is due in part to the release of a 10 kDa peptide from the native protein, by cathepsin-like enzymes present in the insect digestive tract. The entomotoxic peptide, Jaburetox-2Ec, exhibits potent insecticidal activity against several insects, including many resistant to the native ureases. JBURE-I and Jaburetox-2Ec cause major alterations of post-feeding physiological processes in insects, which contribute to, or can be the cause of, their entomotoxic effect. An overview of the current knowledge on plant urease processing and mechanisms of action in insects is presented in this review.

## 1. Introduction

In 1981, a protein named canatoxin (CNTX) was isolated from the seeds of *Canavalia ensiformis *(jackbean) [1]. CNTX was shown to induce convulsions and death in mice and rats when injected intraperitoneally [2], but it was ineffective if given orally to the animals [3]. Twenty years later, CNTX was characterized as an isoform of urease [4]. Ureases (urea amidohydrolase; EC 3.5.1.5) are nickel-dependent enzymes that catalyze the hydrolysis of urea into ammonia and carbon dioxide [5]. These enzymes have been isolated from a variety of organisms, including bacteria, fungi and plants [6]. *C. ensiformis* displays several urease isoforms: jackbean urease (JBURE-I) [7], canatoxin (CNTX) [1,4] and JBURE-II [8,9]. *Glycine max* (soybean) also presents more than one urease: the ubiquitous urease and the embryo-specific urease (herein referred to as SBU) [10,11,12]. Beyond roles related to the catalyzed hydrolysis of urea, ureases were shown to have several properties that are independent of their enzymatic activity [4,13,14]. 

Based on studies with soybean ureases, a role in seed chemical defense, dependent on ureolytic activity has been previously proposed, but an association between ureolysis and plant defense has yet to be demonstrated [15,16]. In the last fifteen years, lines of evidence of an overlooked toxicity of plant ureases toward some insects and fungi—a toxicity independent of enzyme activity—have accumulated [17,18,19,20]. Ureases are members of the arsenal of plant defense proteins, alongside lectins, ribosome inactivating proteins, proteinase inhibitors, amylase inhibitors and arcelins [16,17]. In the current article, an overview of the body of knowledge available to date on the entomotoxic properties of plant ureases is presented.

## 2. The Entomotoxic Activity of Plant Ureases

The widespread presence of “CNTX-like” proteins in other leguminous seeds [21,22], as well as the toxin accumulation pattern during seed maturation [23] strongly suggested that this protein might play an important physiological role, perhaps related to the plant defense. To investigate this hypothesis, the effect of CNTX was studied in different insects (Table 1). CNTX’s entomotoxic properties were first described in 1997 [24]. At that time, little information about the biological properties of CNTX in plants and invertebrates was available. In that study, it became clear that CNTX has target specificity: only insects relying on cathepsin-like enzymes (cysteine and aspartic proteases), such as *Callosobruchus maculatus* and *Rhodnius prolixus*, are sensitive to the toxin, while insects with digestion based on trypsin-like enzymes (serine proteases), such as *Manduca sexta*, *Schistocerca americana*, *Drosophila melanogaster* and *Aedes aegypti*, show no susceptibility. The hypothesis of a proteolytic activation of the toxin was then proposed, and it will be discussed in the next section. 

Later on, it was demonstrated that CNTX has specificity also regarding the life cycle of the target insect: adults of *R. prolixus* are not affected by the toxin, even when higher doses are added to their diets [25]. The same pattern was observed in feeding assays with *Dysdercus peruvianus* and *Nezara viridula*, where nymphs, but not adults, are sensitive to CNTX [17,26]. As will be discussed later, a differential processing of ureases by digestive enzymes in different stages of the insect’s life cycle is potentially involved in the distinct susceptibility of adults and nymphs.

**Table 1:** Entomotoxicity assays with canatoxin (CNTX).

Insect	Insect stage	Administration route	Effect observed	Reference
*M. sexta*	2^nd^ instars	Feeding	None	[24]
*S. americana*	5^th^ instars	Feeding	None	[24]
*D. melanogaster*	Adults	Feeding	None	[24]
*A. aegypti*	Adults	Feeding	None	[24]
*C. maculatus*	Larvae	Feeding	None	[24]
*R. prolixus*	3^rd^ instars	Feeding	Decreased weight loss after feeding	[24]
			80–100% mortality after 72 h	
	3^rd^ instars	Injection	None	[24]
	4^th^ instars	Feeding	65–75% mortality after 48 h	[25]
	Adults	Feeding	None	[25]
	Adults	Injection	None	[25]
*D. peruvianus*	3^rd^ instars	Feeding	Reduced body weight gain	[26]
			Delayed development	
			90–100% mortality after 10 days	
	Adults	Feeding	None	[26]
*N. viridula*	2^nd^ instars	Feeding	100% lethality after 72 h	[17]

CNTX was characterized as a urease isoform only after 20 years of research [4]. This finding raised the question whether the entomotoxic properties displayed by CNTX were shared with other ureases. The major jackbean urease isoform JBURE-I is highly toxic to several insects (Table 2). SBU and JBURE-II also present toxic effects toward insects [9,27], indicating that plant ureases indeed share this activity. On the other hand bacterial ureases, such as the *Bacillus pasteurii* urease, are innocuous to insects [27]. The insecticidal effects of JBURE-I and SBU persist after treatment with an irreversible urease inhibitor, demonstrating that a domain distinct from the active site is involved in the entomotoxic activity [27]. Even though ureases share a high amino acid identity (over 50%) regardless of their source, plant and bacterial ureases have a distinct organization of their subunits, with plant ureases being usually homotrimers or homohexamers of a ~90 kDa subunit, while bacterial ureases are multimers of two or three-subunit complexes [6,28,29]. We know now that the some of the domains involved in the entomotoxic activities of plant ureases are missing in the bacterial ureases [30]. 

**Table 2:** Entomotoxicity assays with jack bean urease (JBURE-I).

Insect	Insect stage	Administration route	Effect observed	Reference
*R. prolixus*	5^th^ instars	Injection	96% mortality after 24 h	[31]
*D. peruvianus*	2^nd^ instars	Feeding	Reduced body weight gain	[27]
			Delayed development	
			85% mortality after 20 days	
	Adults	Feeding	None	
*O. fasciatus*	3^rd^ instars	Feeding	90% mortality after 14 days	[32]

While JBURE-I (0.25 μg/mg of insect fw) is highly toxic when injected into the hemocoel of *R. prolixus* (fifth instar), promoting almost 100% mortality after 24 h [31], CNTX injections into third instars had no effect [24]. On the other hand, only CNTX shows intraperitoneal mammalian toxicity, a property not shared by either JBURE-I or SBU [1,27]. The specific molecular features of these plant urease isoforms that could account for these differences in their toxicity patterns are still unknown.

## 3. The Entomotoxic Peptides

The term “peptide” is used here to emphasize a fragment of a protein, regardless of its molecular mass.

After the first evidence suggesting proteolytic activation of CNTX, many studies were conducted to investigate this hypothesis. CNTX was digested *in vitro* with enzymes obtained from *C. maculatus* larvae, and the resulting peptides were fractionated by gel-filtration and tested for toxicity in nymphs and adult *R. prolixus* [25]. Six peptide pools were originated, and the most toxic effect was observed when insects were fed on Pool C. This fraction, containing peptides of *ca.* 10 kDa, was also toxic to adults, via metathoracic injection, contrasting with the lack of effect observed for the intact protein administrated by the same route [25]. Pools E and F also presented toxicity towards nymphs of *R. prolixus*, indicating that the entomotoxic activity may be associated with a family of peptides, or that the peptides present in Pool C may be further digested, releasing smaller peptides that retain some of the toxic properties. The main peptide found in Pool C, called Pepcanatox, was isolated and sequenced [33]. Further studies on the insecticidal activity and mechanism of action of urease peptides were conducted with a recombinant peptide, derived from the JBURE-II isoform and equivalent to Pepcanatox, named Jaburetox-2Ec [30]. This peptide has 93 amino acids and it is toxic to several insects, including some species that were not affected by the native ureases, such as the fall armyworm *Spodoptera frugiperda* (Table 3). 

**Table 3:** Entomotoxicity assays with Jaburetox-2Ec.

Insect	Insect stage	Administration route	Effect observed	Reference
*D. peruvianus*	3^rd^ instars	Feeding	100% mortality after 11 days	[26]
	Adults	Feeding	None	[26]
*S. frugiperda*	3^rd^ instars	Feeding	Decreased weight gain	[30]
			100% mortality after 8 days	
*R. prolixus*	4^th^ instars	Injection	100% mortality after 48 h	[34]
*T. infestans*	5^th^ instars	Injection	100% mortality after 15 h	[34]
	Adults	Injection	100% mortality after 20 h	[34]

An *ab initio* molecular modeling of Jaburetox-2Ec suggested the presence of structural motifs with characteristics similar to those found in a class of pore-forming peptides [30]. Using large unilamellar vesicles, a simple and convenient model for a biological membrane, it was demonstrated that Jaburetox-2Ec displays membrane-disruptive ability on acidic lipid bilayers [35]. Also, in the same article, it was demonstrated using computational simulations that Jaburetox-2Ec is able to anchor in a polar/non polar interface. This paper presented the first evidence that Jaburetox-2Ec interacts with lipid vesicles and promotes membrane permeabilization. These properties may be relevant to the action of Jaburetox-2Ec *in vivo*. Site-directed mutagenesis studies are now being conducted in order to identify specific regions and amino acid residues involved in Jaburetox-2Ec function.

## 4. Urease Processing by Insect Digestive Enzymes

When an insect is fed on diets containing CNTX plus pepstatin A (an inhibitor of aspartic proteases) or E-64 (an inhibitor of cysteine proteases) the toxic effect is reduced, supporting the idea that CNTX is hydrolyzed by cathepsin-like enzymes in the insects digestive tract [24,25] and that this hydrolysis is a main factor in CNTX’s action in insects. Moreover, that inhibitors of proteases from different classes impair the insecticidal effect of CNTX is a strong indication that the release of entomotoxic peptide(s) from native urease is due to a concerted or sequential action of more than one enzyme.

An effort was made to identify the enzymes responsible for urease proteolytic activation in insects. Midgut homogenates of adults (herein referred to as AH) and 4^th^ instars (herein referred to as NH) of *D. peruvianus* were assayed for their proteolytic activity in the presence of several inhibitors, and some differences were found [26]. Proteases active in acid pH are predominant in both homogenates, although the proteolytic activity shows a shift to higher pH for the adults (maximum activity at pH 3.5–4.0 for NH and at pH 4.5–5.0 for AH). Also, a distinct inhibition profile is observed for each stage: only pepstatin-A inhibits significantly the AH activity, while both E-64 and pepstatin-A inhibit the NH activity. Proteolytic activity at higher pH is only observed in AH (maximum activity at pH 8.5), which could be an indication of the presence of serine proteases in this insect stage. A subsequent study investigated further the processing of ureases by nymphs and adults of *D. peruvianus* [36]. Again, the presence of a serine protease only in AH was evident, since PMSF (phenylmethylsulfonyl fluoride—a serine protease inhibitor) inhibits the azocaseinolytic activity of AH, but not of NH. In this same study, *in vitro* hydrolysis of JBURE-I was performed with both AH and NH, resulting in differential fragmentation of JBURE-I. While NH extensively hydrolyzed JBURE-I producing a ~10 kDa fragment recognized by Jaburetox-2Ec antibodies, in contrast, hydrolysis of JBURE-I by AH does not generate any fragment in the 10 kDa range. Furthermore, the hydrolysis of JBURE-I by AH is reduced in the presence of PMSF, an effect not observed for NH. The presence of this PMSF-sensitive enzyme may cause important differences in the limited proteolysis of urease in the two stages of *D. peruvianus*. Within the entomotoxic peptide sequence [30] there are 11 potential cleavage sites for trypsin. If adults in fact have trypsin-like enzyme(s), it is possible that the entomotoxic peptide is released from urease, but then degraded into smaller, non-toxic, fragments. Another fact supporting the hypothesis that the entomotoxic peptide could be degraded by trypsin-like enzymes is that Jaburetox-2Ec is not toxic to *D. peruvianus* adults when administered orally [26].

Using synthetic substrates that correspond to the *N*- and *C*-terminal regions flanking the entomotoxic peptide within JBURE-I, it was demonstrated that AH has little activity upon the *N*-terminal substrate, indicating that the release of the peptide may not occur in adults. On the other hand, additional data suggested that in nymphs of *D. peruvianus*, a metalloprotease is involved in the limited proteolysis of ureases and release of the entomotoxic peptide. The *in vitro* hydrolysis of JBURE-I by midgut homogenates of nymphs of *Oncopeltus fasciatus* also produces a peptide of ~10 kDa that is recognized by Jaburetox-2Ec antibodies [37]. Since no metalloprotease activity was detected in midgut homogenates of this insect, it is possible that another class of enzyme is involved in *O. fasciatus* in the limited proteolysis of ureases. Another alternative is that the enzymatic cleavage sites within the urease sequence may differ according to the susceptible insects, generating entomotoxic peptides with slightly different sequences, butthat retain entomotoxic activity.

It is clear that the differences in urease susceptibility of adults and nymphs of different insects are complex, with the contribution of multiple factors, including a stage-specific release/processing of the entomotoxic peptide(s) from the intact protein and possibly the transport of the urease/peptide(s) from the lumen of the gut into the hemolymph, where it can reach the targets of the toxic effect.

## 5. Targets of Action

In mammalian models (rats, mice and rabbits), CNTX induces exocytosis in platelets, mast cells, brain synaptosomes, isolated pancreatic islets, neutrophils and macrophages, but without altering the cell membrane integrity [38,39,40,41,42]. Many of these activities are shared by other ureases from plants and bacteria [14,27,43]. On the other hand, little is known about the targets of action of ureases in insects. In recent years, two insect systems have been shown to be affected by JBURE-I and Jaburetox-2Ec.

### 5.1. Malpighian Tubules

During each instar, *R. prolixus* can consume a blood meal equivalent to 10–12 times its initial body weight, and it is essential that much of this fluid load be voided as fast as possible. To achieve this, the insect starts to urinate even before it has finished the blood meal, and during the first 24 h after feeding, the insect eliminates over 40% of the weight of the blood meal as dilute urine [44]. 

A decrease in body weight loss is detected in *R. prolixus *after feeding on CNTX, suggesting an impairment of water balance [24]. To further investigate if ureases could in fact impair urine production in *R. prolixus*, a modified Ramsay assay was employed, in order to evaluate urine secretion in Malpighian tubules [45]. JBURE-I, at doses as low as 5 × 10^−14^ M, is able to inhibit the serotonin-induced secretion, while the highest inhibition is observed with 5 × 10^−10^ M JBURE-I (considering the urease hexamer molecular mass). CNTX inhibits secretion at the same level as JBURE-I, but the urease from the bacteria *Helicobacter pylori* is not as potent as JBURE-I and CNTX at the tested doses. Jaburetox-2Ec is also inhibitory to the Malpighian tubules secretion, at doses even lower (10^−16^ and 10^−15^ M) than those effective for JBURE-I [45]. In this same study, the possible second messengers involved in JBURE-I and Jaburetox-2Ec were investigated and data obtained showed that inhibition of secretion occurred through different mechanisms.

Jaburetox-2Ec increases cGMP levels in the tubules in the presence of serotonin. cGMP is a second messenger to some antidiuretic factors in *R. prolixus* [46,47], where it potentially blocks the apical V-ATPase found in the Malpighian tubules cells [45]. 

Several effects observed for CNTX and JBURE-I in mammalian systems are mediated/modulated by eicosanoids [13,14,48], and it seems that JBURE-I is acting on the Malpighian tubules also through the eicosanoid metabolite pathway, since its activity is reversed by dexamethasone (an indirect inhibitor of phospholipases) and is influenced by intra- and extracellular calcium [45]. Since PGE_2_ is able to inhibit fluid secretion in *R. prolixus *Malpighian tubules [45], it is possible that this is the eicosanoid metabolite that acts as second messenger for JBURE-I. The target to PGE_2_ in the tubules is still unknown, but in rat kidneys, PGE_2_ stimulates removal of aquaporins from the surface of the principal cells [49]. Aquaporins have been described in *R. prolixus* Malpighian tubules [50] and demonstrated to be modulated by serotonin [51], therefore aquaporins could be considered a possible target for JBURE-I action. 

Recent results suggest that the inhibitory actions of urease isoforms on *R. prolixus* Malpighian tubules are likely independent of the signaling cascade involving RhorpCAPA-2, an antidiuretic hormone, and its cognate receptor, RhoprCAPAr1, since the inhibition of Malpighian tubule fluid secretion by this hormone is independent of eicosanoid metabolites and calcium [52].

### 5.2. Anterior Midgut

*R. prolixus* anterior midgut is also involved in the post-feeding diuresis, and could be a target for urease action as well. Several assays were performed with this tissue, in order to evaluate JBURE-I effects [31]. As described for *Bombyx mori* [53,54] it was found that the intact active JBURE-I can pass through the anterior midgut epithelium into the hemolymph of *R. prolixus*, which indicates that the toxin can reach several tissues in the insect after its ingestion, and potentially interfere on different physiological processes. In *B. mori* it was suggested the presence of a urease binding molecule in the brush border membrane of the insect midgut epithelium, which could be involved in the transport of the mulberry leaf urease [55]. To this date, we have no data concerning urease transport mechanism in *R. prolixus*.

JBURE-I causes a dose-dependent decrease in serotonin-induced fluid absorption when injected into the lumen of the anterior midgut of *R. prolixus*, but no changes were observed on ion transport (short circuit current, transepithelial voltage and resistance of the tissue were unaltered) [31]. Also, JBURE-I potentiates the frequency of serotonin-induced contractions, in a dose-dependent fashion, and increases the strength of those contractions. However, JBURE-I does not affect spontaneous contractions of the anterior midgut. As demonstrated for Malpighian tubules, eicosanoid metabolites, more specifically prostaglandins, mediate the effects of JBURE-I in the anterior midgut. 

### 5.3. Other Possible Targets

Considering the results obtained with Malpighian tubules and the anterior midgut [31,45], it became clear that JBURE-I interferes with processes coordinated by serotonin in insects. Contractions of the hindgut, which aid in urine expulsion and in mixing gut contents as well as mixing the local hemolymph, are also under control of serotonin [44]. JBURE-I has a similar effect on the hindgut as observed in the anterior midgut—it increases the frequency and the amplitude of the serotonin-induced contractions [56]. This effect can also contribute to an altered physiology in insects fed with ureases. 

Several other tissues have their function regulated by serotonin in insects, such as salivary glands, heart and dorsal vessel. Evaluation of the effect of ureases and their peptides in these other tissues are crucial to help to elucidate the complete mechanism of action of these toxins. 

## 6. Conclusions

**Figure 1:**
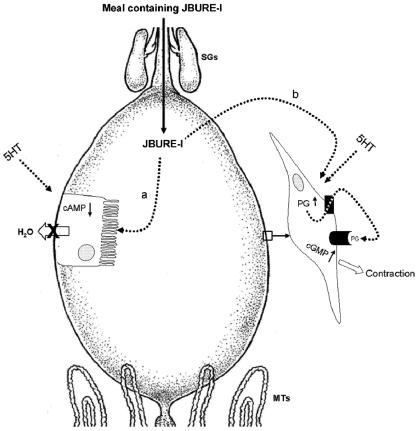
Proposed model for urease action on the anterior midgut. During feeding, serotonin is released into the hemolymph and JBURE-I is found in the lumen of the anterior midgut, acting on epithelial cells (**a**), causing a decrease in serotonin-stimulated cAMP levels and disrupting the fluid transport across the epithelium. After 30 min, the transport of JBURE-I into the hemolymph has started, where it can act on muscle fibers (**b**), promoting an increase in PGs levels, which leave the cell by the action of a PG transporter and then interact with G-protein linked receptors, increasing the concentration of cGMP. Increased levels of cGMP potentiate the frequency of serotonin-induced contractions. SG: salivary glands; AMG: anterior midgut; MTs: Malpighian tubules; 5HT: serotonin; PGs: prostaglandins. Adapted from [31].

**Figure 2:**
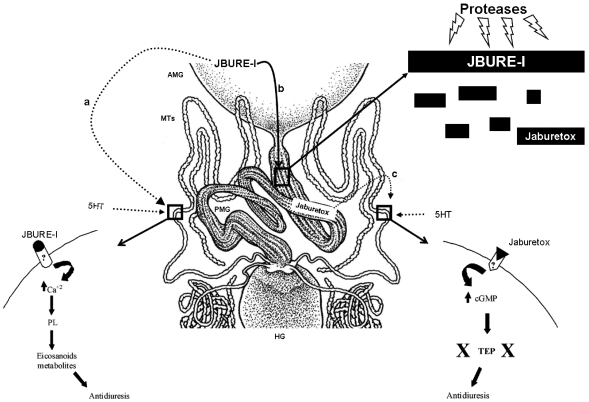
Proposed model for urease and Jaburetox-2Ec action on Malpighian tubules. In the hemolymph, JBURE-I can act on the Malpighian tubules (**a**), where it disrupts the diuresis via eicosanoid metabolites. JBURE-I that remains in the anterior midgut is then transported to the posterior midgut, where it is processed by the insect digestive enzymes, releasing several peptides, including Jaburetox (**b**). Jaburetox reaches the hemolymph, where it interferes with diuresis in the Malpighian tubules by disrupting the transepithelial potential (**c**). PGs: prostaglandins; PL: phospholipase; AMG: anterior midgut; PMG: posterior midgut; MTs: Malpighian tubules; HG: hindgut; 5HT: serotonin; TEP: transepithelial potential. Adapted from [57].

The available data concerning urease actions in insects are summarized in Figure 1 and Figure 2. Urease toxicity to insects is a complex event, involving the intact protein and peptides released by the action of insect digestive enzymes. Over the last years, an increasing amount of information about this subject was generated, shedding light onto the mechanism of action of ureases in insects. Possible targets within the insects were identified and the molecular pathways triggered by this class of toxins began to be elucidated. Nevertheless, we still have a long way in front of us before we can completely understand the many effects of these proteins, from the moment the insect encounters the toxin until the insect death. Also, there is a need for adequate understanding of insect physiology when considering the potential biotechnological use of insecticidal proteins in protecting crops against insect pests. Plant ureases and their derived peptides have a great biotechnological potential. Exploring the entomotoxic properties of these molecules will be important for the development of alternative strategies to protect commercially-relevant crops against natural enemies.
